# On the brink of extinction: the future of translational physician-scientists in the United States

**DOI:** 10.1186/s12967-017-1188-6

**Published:** 2017-05-01

**Authors:** Hideki Furuya, Dean Brenner, Charles J. Rosser

**Affiliations:** 1 Clinical and Translational Research Program, University of Hawaii Cancer Center, 701 Ilalo St, Rm 327, Honolulu, HI 96813 USA; 2 Division of Hematology-Oncology, Department of Internal Medicine, University of Michigan School of Medicine, Ann Arbor, USA

**Keywords:** Translational research, Physician-scientist, Career, Funding, NIH

## Abstract

Over the past decade, we have seen an unparalleled growth in our knowledge of cancer biology and the translation of this biology into a new generation of therapeutic tools that are changing cancer treatment outcomes. With the continued explosion of new biologic discoveries, we find ourselves with a limited number of trained and engaged translational physician-scientists capable of bridging the chasm between basic science and clinical science. Here, we discuss the current state translational physician-scientists find themselves in and offer solutions to navigate during this difficult time.

Over the past decade, we have seen an unparalleled growth in our knowledge of cancer biology and the translation of this biology into a new generation of therapeutic tools that are changing cancer treatment outcomes. With the continued explosion of new biologic discoveries, we find ourselves with a limited number of trained and engaged translational physician-scientists capable of bridging the chasm between basic science and clinical science.

Why is this happening? Adverse financial pressures on institutions with tripartite missions of research, patient care and education, specifically, Universities and their associated hospitals, due to political, socio-economic and cultural changes, appear to be the root cause. First, to compensate for reduced medical reimbursements associated with managed care and reduced payments through government supported and mandated coverages, institutional managers have pressed clinicians to increase patient throughput—see more patients with less time per patient. Second, in order to sustain their research mission, physician-scientists require more resources (i.e, clinical and administrative support) than their non-scientist counterparts. Third, the rise of for-profit hospitals has siphoned resources from the tripartite missions to excessive executive salaries, to shareholders, to Wall Street investors and to highly paid consultants.

Prior financial models enabled cost shifting of hospital surpluses to the academic mission of the University. This model has disintegrated because (a) hospital margins are eroding due to higher expenses and lower reimbursements; (b) heavy bureaucratic costs to meet increasingly complex regulations; and (c) an influx of newly but insufficiently covered patients through the Accountable Care Act. External funding agencies, primarily the NIH, have not been able to allocate sufficient resources to training and early career development support. As a result of these institutional revenue losses combined with reductions in NIH funding resources, sources for salary support and resources for physician-scientists have become scarce.

Early career physician-scientists, specifically physician-scientists, appear to be the most vulnerable. A review of funded early physician-scientist Grants (e.g, K08 and K23) from NCI has demonstrated a persistent decline in the number of grants funded since 2007 (Fig. 1). The decline in early career physician-scientist funding coincides with the Great Recession of 2008. In the economic recovery that has followed from 2013 to the present, NIH has not reinstated physician-scientist funding to previous levels. Fewer physician-scientist grants are being submitted to NCI.

**Fig. 1:**
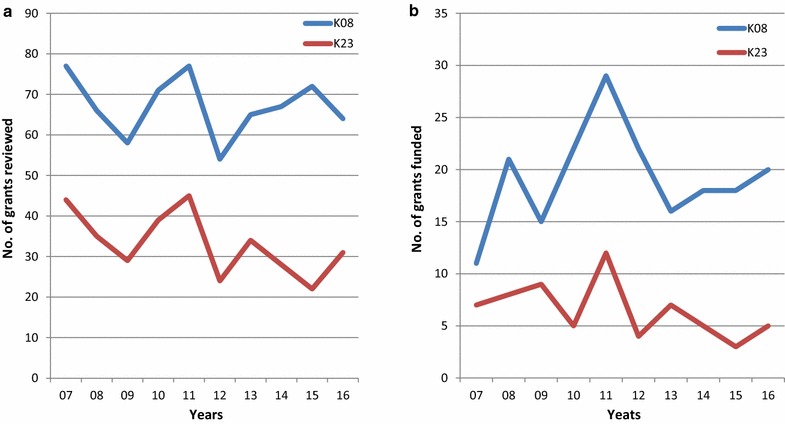
K08 and K23 NCI Grants **a** reviewed and **b** funded from 2007–2016

In 1981, Dr. James Wyngaarden (NIH Director 1982–1989) first reported the decline in the number of physician-scientists [1]. In this report, he describes the decline in awards to develop physician-scientists. Beginning 40 years ago, in the 1970s, physician-scientists were being replaced by Ph.D. scientists specializing in ‘clinical research’ [1]. This trend continues. While we recognize the importance of Ph.D. scientists in filling the gap in translational research, they are not trained in the breadth and nuances of clinical practice, especially when the landscape of clinical practice is changing everyday.

In 2005, a Blue Ribbon panel of the National Academy of Sciences reported the decline in funding of young scientists. In 1983, 18% of R01 awardees were <36 years of age. By 2010, <3% of R01 awardees were <36 years. In addition, the panel noted the departure of young minds from the field [2, 3]. In 2014, a report from a panel commissioned by NIH to assess physician-scientist workforce was published. The report noted a reduction in the physician-scientist workforce possibly to due to challenges confronting the physician-scientist. These challenges include increased costs of medical education, training required for competency in clinical and scientific research, funding pressures, financial opportunities in clinical practice, balancing work/life demand and finding a suitable mentor [4].

Based on these reports, NIH adopted the following reforms: expand awards for young investigators, expand mentoring opportunities, easing requirements of preliminary data for young investigators applying for grants, diversifying study review panels and improving career paths and enhance data collection. Despite these reforms, physicians interested in careers in research have continued to wane over the past 10 years.

The public, represented by government agencies, demands rapid translation of scientific discovery. The Cancer Moonshot Initiative, a great example of public pressure being implemented as policy, will be seriously handicapped without the necessary manpower to translate the science to practical clinical application. To meet this need, we suggest that policies and resource allocation will need to be reconsidered at all levels within the system—at the NIH, the ACGME, AAMC, the Department of Veterans Affairs, the National Academy of Science, and key societies (e.g. ASCO and AACR) should examine the root cause of our current physician-scientist workforce problem. A concerted, focused program requires commitments from all relevant stakeholders with the goal of providing sufficient resources to enable committed and capable junior faculty sufficient time, space, and resources to succeed. Among the potential resources that would need to be enhanced, further expansion of training grants at the entry level (K99/R00, K07, 08, 23). Universities will need to cost shift resources from clinical revenue and overhead revenue to cover salary shortfalls in the early years of faculty appointment. Philanthropic funding is likely to play a crucial future role in support of the junior physician-scientist. Creation of endowed funds aimed at time-limited salary support for entry-level junior faculty would enable the necessary protected time and reduce the pressures for immediate income generation.

The physician-scientist model, unique to the United States’ leadership in biomedical research, is endangered. If we fail to find novel solutions to this “brain drain”, we will lose our competitive advantage as a country and more importantly, our patients will suffer. Now more than ever, we need translational physician-scientists to assist in (a) identifying key clinical barriers that can be addressed through research and (b) bridging the ever increasing gap of data that stands to hold key discoveries to ensure the health of our patients. The departure of translational physician-scientists from the academic biomedical workforce in turn poses grave risks for the future of medicine.

## Abbreviations

NIH: National Institutes of Health
NCI: National Cancer Institute
ACGME: Accreditation Council for Graduate Medical
AAMC: Association of American Medical Colleges
ASCO: American Society of Clinical Oncology
AACR: American Association for Cancer Research
